# Integrated telehealth intervention to reduce chronic pain and unhealthy drinking among people living with HIV: protocol for a randomized controlled trial

**DOI:** 10.1186/s13722-024-00493-3

**Published:** 2024-09-05

**Authors:** Tibor P Palfai, Lauren B Bernier, Maya PL Kratzer, Kara M Magane, Sarah Fielman, John D Otis, Timothy C Heeren, Michael R Winter, Michael D Stein

**Affiliations:** 1 Department of Psychological and Brain Sciences, Boston University, 900 Commonwealth Ave, 2nd Floor, Boston, MA 02215 USA; 2 Department of Community Health Sciences, Boston University School of Public Health, Boston, MA USA; 3 Biostatistics and Epidemiology Data Analytics Center, Boston University School of Public Health, Boston, MA USA; 4 Department of Health Law, Policy and Management, Boston University School of Public Health, Boston, MA USA

**Keywords:** Alcohol, Chronic pain, HIV, Telehealth, Ecological momentary assessment

## Abstract

**Background:**

Unhealthy alcohol use represents a significant risk for morbidity and mortality among people living with HIV (PLWH), in part through its impact on HIV management. Chronic pain, a common comorbidity, exacerbates suboptimal engagement in the HIV care continuum and has reciprocal detrimental effects on alcohol outcomes. There are no integrated, accessible approaches that address these comorbid conditions among PLWH to date. This paper describes a research study protocol of an integrated telehealth intervention to reduce unhealthy drinking and chronic pain among PLWH (Motivational and Cognitive-Behavioral Management for Alcohol and Pain [INTV]).

**Methods:**

Two-hundred and fifty PLWH with unhealthy drinking and chronic pain will be recruited nationally via online advertisement. Informed consent and baseline assessments occur remotely, followed by 15 days of ecological momentary assessment to assess alcohol use, chronic pain, functioning, and mechanisms of behavior change. Next, participants will be randomized to either the INTV or Control (CTL) condition. Individuals in both conditions will meet with a health counselor through videoconferencing following randomization, and those in the INTV condition will receive 6 additional sessions. At 3- and 6-months post-baseline, participants will complete outcome assessments. It is hypothesized that the INTV condition will result in reduced unhealthy alcohol use and pain ratings compared to the CTL condition.

**Conclusion:**

This protocol paper describes a randomized controlled trial which tests the efficacy of a novel, integrated telehealth approach to reduce unhealthy alcohol use and chronic pain for PLWH, two common comorbid conditions that influence the HIV treatment cascade.

**ClinicalTrials.gov identifier:**

NCT05503173.

## Highlights

Unhealthy alcohol use and chronic pain are common in people living with HIV.

An integrated telehealth intervention may improve alcohol, pain, and HIV outcomes.

Ecological momentary assessment will examine processes underlying behavior change.

## Introduction

Unhealthy alcohol use represents a significant risk for morbidity and mortality among people living with HIV (PLWH) and is estimated to occur among 10 to 38% of patients depending on sample characteristics [1–3]. Unhealthy alcohol use refers to the consumption of alcohol that places individuals at risk for negative health outcomes and ranges from consumption of amounts that represent increased risk through severe alcohol use disorders [4]. Heavy drinking, defined as consuming more than 14 standard drinks per week or more than 4 standard drinks on any day for men and more than 7 standard drinks per week or more than 3 standard drinks on any day for women, is commonly identified as an amount that represents risk to health [5]. However, the amount of alcohol associated with mortality and physiologic injury may be lower among PLWH [6]. Both suboptimal viral suppression and faster HIV disease progression are associated with unhealthy alcohol use, [5, 7] which may be mediated directly through biological mechanisms (e.g., inflammatory processes) or indirectly through engagement in a variety of behaviors including poor linkage to care, missed healthcare appointments, suboptimal or non-adherence to antiretroviral therapy (ART), unprotected sexual activity, and injection drug use [1, 8–11]. Unhealthy alcohol use is also associated with the likelihood and severity of several comorbid conditions among PLWH impacting mental and physical health [12, 13]. 

Common comorbidities among PLWH, including chronic pain, exacerbate unhealthy drinking and its impact on HIV outcomes. Among patients presenting to HIV clinics, 35–50% experience chronic pain [12, 14]. Moreover, as the population of PLWH ages, pain has become more common, originating as a complication of HIV itself (e.g., neuropathy; [15]) and from common age-related conditions (e.g., arthritis). Not only is chronic pain associated with more severe HIV-related symptoms and reduced engagement with HIV care [16], but it also increases negative alcohol-related consequences among patients meeting criteria for unhealthy drinking across medical settings [17, 18]. Pain influences alcohol use through a variety of pathways (e.g., alcohol use to cope with pain; [19]) and compounds the impact of unhealthy alcohol use on HIV-related health outcomes such as frailty and poor physical functioning [20–22]. Given that pain commonly co-occurs with unhealthy drinking among PLWH, that they have reciprocal influences on one another [17], they impact mental and physical functioning, and they have additive effects on HIV-disease progression [14, 23], it is critical to develop and test approaches to address these concerns among this population.

While pharmacological treatments are available for pain, they are often inadequate for long-term pain management and complicated by comorbid conditions, including substance use history [24]. It is therefore often necessary to identify effective non-pharmacological treatments to adequately manage pain over time. Efforts to address unhealthy drinking among PLWH present other challenges, as the majority of individuals are unaware of the health risks associated with their alcohol use and do not seek alcohol treatment resources [25]. Furthermore, there are few alcohol services routinely offered in HIV care settings [26]. The need for multiple in-person clinic visits compounds challenges related to access, utilization, and adherence to behavioral treatments for unhealthy drinking and pain. Providing alternative intervention modalities that do not require repeated in-person medical visits is critical for reducing patient burden, increasing access to care, and improving treatment efficacy.

This paper describes a study protocol for a randomized controlled trial (RCT) that aims to test the efficacy of an integrated telehealth intervention for reducing unhealthy drinking and chronic pain among PLWH that may be implemented in a fully remote context. The secondary aims are to examine the moderators of the Motivational and Cognitive-Behavioral Management for Alcohol and Pain (INTV) effect and the mediational pathways of the INTV effect. Baseline interview-administered self-report and ecological momentary assessment (EMA) will be used to identify potential individual difference factors that moderate intervention efficacy. Post-intervention EMA, which provides the advantage of real-time assessment of self-regulatory processes and contextual influences on those processes, will be used to identify potential mediators of the intervention and explore mechanisms of behavior change [27, 28].

## Method

This RCT is a project component of ARCHER (Addressing Related Comorbidities for HIV by Employing Remote technologies), an HIV Center funded by National Institute on Alcohol Abuse and Alcoholism (P01AA029546). The trial is registered on ClinicalTrials.gov (NCT05503173). This study is conducted in compliance with the protocol and applicable regulatory requirements approved by the Boston University Medical Campus/Boston Medical Center Institutional Review Board and Human Research Protection Program.

### Overall design

**Fig. 1:**
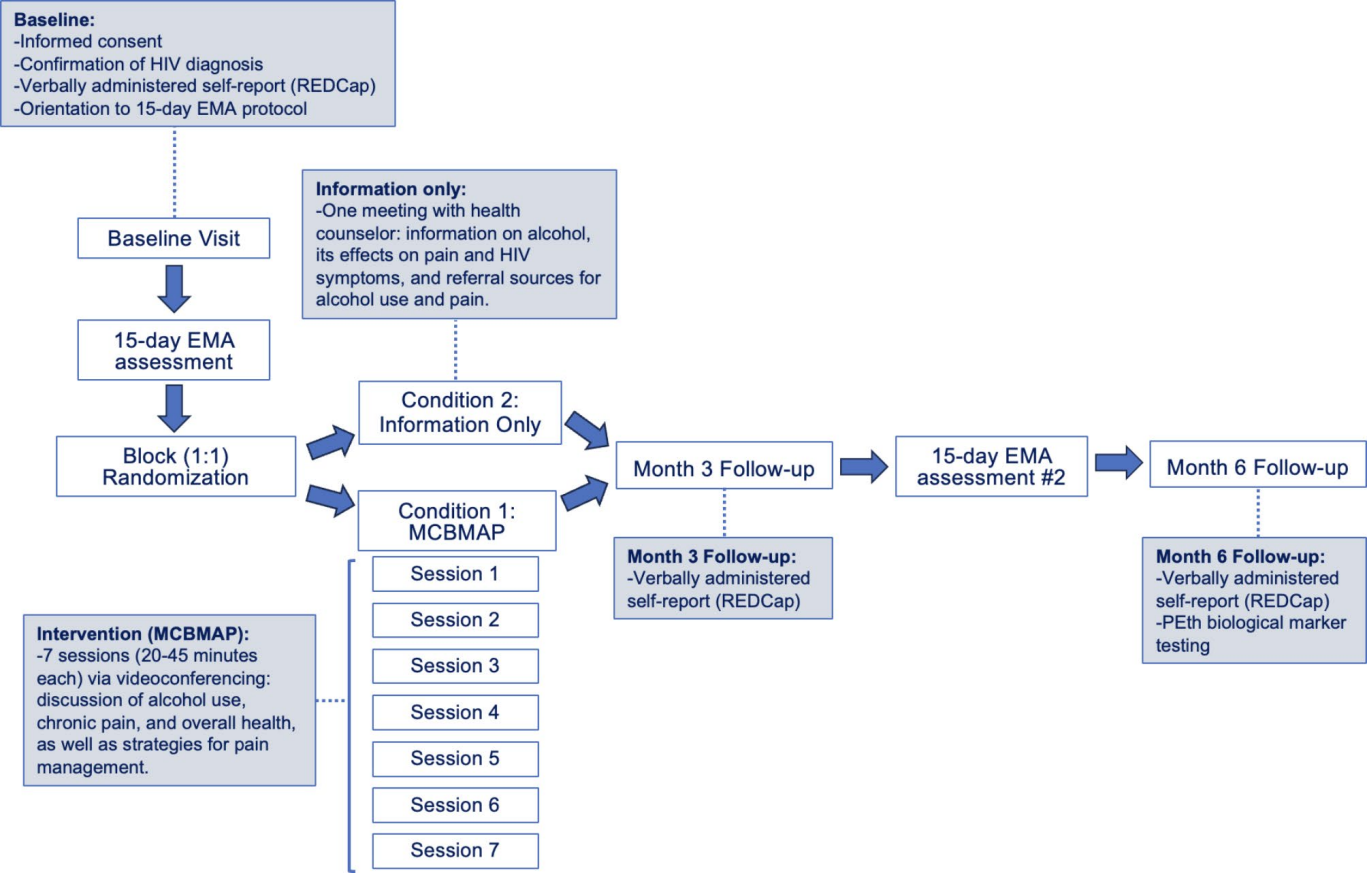
Study flow diagram

Participants will be PLWH who engage in unhealthy drinking and experience moderate or greater chronic pain recruited nationally via online advertisement. Those who are eligible based on an initial set of online prescreening questions will be contacted by study staff for a more comprehensive phone screening. Those who remain eligible based on the more extensive screening will be scheduled for baseline assessment during which they will provide informed consent, confirm HIV diagnosis, and complete baseline questionnaires administered in an interview format. Staff will also orient participants to the EMA procedures whereby they will complete 5 surveys per day for the following 15 days. After EMA data collection, participants will undergo 1:1 randomization into either the intervention group (INTV) or the control (CTL) group. The INTV condition is a 7-session program designed for weekly delivery through videoconferencing by a health coach. Those in the CTL group meet virtually with the health coach for a single session and be provided with information and resources about alcohol and pain. At three-month outcome assessment, staff will administer questionnaires via interview, prior to another 15 days of EMA data collection. The final study assessment occurs six months after randomization and will include a staff-administered interview and a self-collected blood draw to measure phosphatidylethanol (PEth), a biological marker of alcohol use (see [29]). Participants complete self-collected blood draws at home and with guidance through videoconferencing (Fig. 1).

### Rationale for study design

The study is designed to test the efficacy of INTV, but has several additional objectives including, (1) the use of a nationally representative sample, (2) implementation of all study procedures remotely, (3) being able to identify potentially important moderators of intervention efficacy, and (4) providing detailed analyses of the processes through which the intervention may impact outcomes. Recruitment through social media will target PLWH across the country in areas defined by the CDC as having high HIV prevalence rates. This fully remote approach reduces participant burden and increases study efficiency. Finally, the study design incorporates the use of EMA. Multilevel modeling allows for an examination of how treatment may change associations between contextual cues (e.g., mood) and outcomes of interest (e.g., drinking, pain) over time. This approach enables fine-grained analysis of how the intervention may impact pain, alcohol use, and their interaction, in addition to providing insight into psychological processes (e.g., self-efficacy) through which these influences may occur. Such information will provide insight into how and for whom the interventions may reduce heavy drinking and pain.

### Study aims and hypotheses

#### Primary aim

This study aims to test the efficacy of INTV for reducing unhealthy drinking and chronic pain among PLWH. The primary alcohol outcomes will be the average number of drinks per week and number of heavy drinking episodes over the past month. The primary pain outcome will be a composite measure of pain intensity and interference (i.e., pain average, interference with enjoyment of life, and interference with general activity) known as the PEG; [30]). Primary analyses will focus on alcohol and pain outcomes assessed at 6-month follow-up. It is hypothesized that those randomized to INTV will show fewer drinks per week, fewer episodes of heavy episodic drinking (primary alcohol outcomes), and lower ratings of pain at the 6-month assessment compared to the those in the CTL group, controlling for corresponding baseline values.

#### Secondary aims

Secondary aims seek to identify potential moderators and mediators of INTV, using data from assessment interviews and EMA. It is hypothesized that the influence of the intervention will be moderated by baseline readiness-to-change (for alcohol) and baseline GSAB questionnaire ratings of self-efficacy and planning (for both alcohol and pain outcomes). Secondary aims pertaining to alcohol use will be addressed by using baseline interview measures and parallel analyses will be conducted using baseline daily EMA ratings of readiness-to-change, alcohol coping self-efficacy, and alcohol-related planning. Mediational analyses will also be conducted using EMA at the 3-month outcome to test hypothesis that changes in self-regulatory processes mediate the effect of the INTV on alcohol use and chronic pain.

### Inclusion and exclusion criteria

Inclusion criteria will consist of the following: (1) at least 18 years of age; HIV + with confirmation via visual evidence of antiretroviral (ART) medication or medical record; (2) engaged in unhealthy drinking defined as: a) having more than 7 drinks for women or 14 drinks for men per week; or b) using an HIV specific index of heavy episodic drinking, having 3 or more drinks for women or 4 or more drinks for men on one occasion in the past month [31–33]; (3) experiencing moderate or greater chronic pain (a score of 4 or greater on the Numerical Pain Rating Scale [NPRS]) for at least three months per self-report); (4) own a smart phone; and live in the United States with a United States mailing address. Exclusion criteria are as follows: (1) a history of bipolar disorder, schizoaffective disorder, or schizophrenia per self-report; (2) an unstable dose of psychoactive medication for pain or alcohol/substance use [i.e., if on medication, participant has not been on same dose for least 2 months]; (3) a history of withdrawal-related seizures or delirium tremens per self-report; current non-pharmacological treatment for alcohol use disorder or chronic pain per self-report; (4) acute life-threatening illness that requires treatment or intention to have surgery for a pain-related condition in the next 6 months; (5) current cancer-related pain per self-report; (6) unwilling to provide sex at birth; (7) limited or non-reader; or (8) unable to provide one or more contacts to facilitate follow-up in case the participant cannot be reached.

### Recruitment, consent, and randomization

Recruitment will take place nationally via online advertisement in virtual patient communities, Google, Facebook, Instagram, health websites, and medical applications. BuildClinical (BC), a company that delivers study specific advertisements to various online platforms, regularly monitors metrics (i.e. percentage of potential participants who view and subsequently click an advertisement) to assess effectiveness and optimize the campaign to improve reach as necessary. When potential participants click on a study advertisement, a website populates containing general information about the trial and a link to a BC pre-screening form for those who are interested. BC pre-screening forms ask for contact information (name, e-mail, telephone number) and assess a preliminary subset of study eligibility criteria (e.g., HIV status, drinking habits, pain level).

If potential participants are eligible based on their responses to the preliminary BC pre-screening, research staff will contact the individual via phone call to confirm pre-screening questions and ask additional eligibility questions. At the time of consent, study staff will visually confirm HIV diagnosis via medical record or ART medication. Eligible and interested participants will take part in an informed consent process via videoconference or phone call and those who provide their consent electronically sign a REDCap [34, 35] form.

Randomization occurs after the baseline assessment phase is completed (described below) and is stratified by both gender and frequency of heavy drinking episodes. The project statistician will generate a list of randomization assignments to be used sequentially based on a permuted blocks strategy.

### Assessments

#### Assessment procedures

Assessments will include interview-administered instruments at baseline, 3 months post-randomization, and 6 months post-randomization. In addition, there will be two 15-day EMA data collection periods (one at baseline and one at 3-months), a treatment satisfaction measure, and a blood draw to test a biological marker of alcohol use, phosphatidylethanol (PEth; [29]) self-administered 6 months-post randomization.

Participants will complete all assessment interviews (baseline, 3-month follow-up, and 6-month follow-up) virtually through a videoconferencing platform or by phone. Interviews will take approximately 90 to 120 min and are each associated with $50 compensation for time and effort.

In addition to research interviews, participants will complete two phases of EMA data collection over 15-day periods. The first 15-day EMA period begins following the baseline interview while the second 15-day EMA period takes place following the 3-month assessment interview. Participants will learn about EMA procedures following the baseline interview, including how to use the EMA iOS or Android mobile application (MetricWire; [36]. Each day during the EMA periods, MetricWire will prompt participants to complete a morning survey and four additional surveys which arrive randomly throughout the day.

Upon completing the 15 days of EMA surveys at the 3-month time point, participants will be notified to complete one final survey on intervention satisfaction and acceptability for which they will be compensated $5. Across all study activities, participants will be able to earn up to $480 over the 6 months. Payments are made via electronic gift certificates.

Finally, at the 6-month outcome assessment interview, participants will be able to earn an additional $25 for completing and sending a self-administered blood sample to the collaborating laboratory [29].

#### Assessment instruments

##### Sociodemographic characteristics

Sociodemographic information will be collected during screening and baseline interview and includes the following: age, state and zip code, sex assigned at birth, gender identity, height, weight, sexual orientation and whom they have sex with in their life (men only, women only, both men and women, not had sex), marital status, current spouse/partner, ethnicity, race, primary language, education, employment, insurance, difficulty paying bills, and unhoused status.

##### Alcohol assessment measures

The primary outcomes for alcohol use include number of alcoholic drinks consumed per week and number of heavy episodic drinking days in the past month (assessed via Alcohol Timeline Followback – 30 days [TLFB-30]; [37, 38]). Alcohol-related consequences will also be assessed using the Short Inventory of Problems-Revised (SIP-R; [39]) as a secondary outcome. A series of measures related to the self-regulation of alcohol use will also be administered, including the Readiness to Change Questionnaire (RTCQ; [40] and Goal Systems Assessment Battery (GSAB; [41, 42]), which provides assessment of value, self-efficacy, self-monitoring, and planning related the goal of moderating alcohol use.

##### Pain assessment measures

The primary outcome for pain is a composite measure of pain severity and interference (pain average, interference with enjoyment of life, and interference with general activity [PEG]; [30]). More comprehensive indices of pain intensity and pain interference will also be assessed using the Brief Pain Inventory- Short Form (BPI; [43]). The GSAB for pain will also be administered to assess self-regulatory components related to the goal of pain management, specifically planning and self-efficacy [41, 42].

##### EMA

As noted above, participants will complete two 15-day periods of EMA: one following the baseline interview and one following the 3-month interview assessment. Each day, participants will complete a morning survey and a set of 4 shorter random surveys. Compensation for completing EMA surveys is $3 for each morning survey and $1 for each random survey. Those who complete all 5 EMA surveys on any given day receive a $3 bonus. Participants can earn up to $10 per day for completing EMA surveys, with a maximum total of $150 per 15-day EMA span ($300 for both timepoints). EMA surveys were modified from previous EMA studies (e.g., [27, 44, 45]) to include questions about alcohol use [46, 47], chronic pain [30], physical and mental functioning [48–50], affect [51, 52], sleep [53], other substance use (i.e., cannabis and tobacco; [54, 55]), and self-regulatory processes [41, 42] that underlie pain management and drinking. EMA data will be collected via MetricWire, a smartphone application that is downloaded onto participants’ phones. Participants will be given the option to use their own email and private password or use a study provided email and password to access the program. The EMA surveys will consist of two assessment types, a morning survey and random survey. Morning surveys will be available from 4am to 10:30am and are initiated by the participant to report aggregate experiences over the past 24 h. Two reminders will be sent within that block to confirm the availability of the morning survey which will take approximately 5 min to complete. Random surveys are signal-contingent recordings whereby participants respond to prompts about their current pain, physical activity, alcohol and other substance use, and affect. They occur randomly in 3-hour blocks (e.g., 9am-noon; noon-3pm; 3-6pm; 6-9pm) and will take approximately 3 min to complete.

##### Phosphatidylethanol

PEth biomarker tests will be used as a marker of unhealthy alcohol use, and is an additional outcome to supplement self-report outcome measures [56]. Blood samples will be collected by the participants using a Tasso-M-20 device [57] with guidance from research assistants. The device uses a button that sticks to the skin, typically on the shoulder. When the instrument is pressed, a lancet pierces the skin and draws blood from the capillaries into a container at the bottom of the device. This procedure produces little discomfort and can be used with minimal training.

The full set of assessments is presented in Table 1.

**Table 1:** Enrollment, intervention, and assessment schedule

TIMEPOINT	Enrolment	Baseline	Randomization	Month 3	Month 6
**Enrolment**					
Eligibility Screen	X				
Informed Consent	X				
Allocation			X		
**Intervention**					
Motivational and Cognitive-Behavioral Management for Alcohol and Pain (INTV) *or*				X	X
Information-Only Control (CTL)				X	X
**Assessments**					
Sociodemographic Characteristics		X			
Current Medications, Medication History		X		X	X
HIV Viral Load		X			X
Treatment Induced Neuropathy Assessment Scale [58]		X		X	X
Alcohol Timeline Followback – 30 days^†^ [37, 38]		X		X	X
Short Inventory of Problems – Revised, Alcohol^†^ [39]		X		X	X
Alcohol Use Disorder Identification Test (Consumption Questions)^†^ [59, 60]		X		X	X
Social Network Drinking^†^ [61]		X		X	X
Addiction Severity Index – 30-day illicit drug use^†^ [62]		X		X	X
CDC Smoking and Vaping Assessment [63]		X		X	X
Alcohol, Smoking and Substance Involvement Screening Test^††^ [64]		X		X	X
Pain, Enjoyment of life, and General Activity assessment^§^ [30]		X		X	X
Brief Pain Inventory – Short Form^§^ [43]		X		X	X
Physical Activity Questionnaire^§^ [65]		X		X	X
International Physical Activity Questionnaire^§^ [66]		X		X	X
Goal Systems Assessment Battery – Pain^§,††^ [41, 42]		X		X	X
HIV Symptom Index [67]		X		X	X
HIV Risk Behaviors [68]		X		X	X
Berger HIV Stigma Scale [69]		X		X	X
Discrimination and Stress Scales [70–72]		X		X	X
Modified Positive and Negative Affect Schedule^¶^ [51]		X		X	X
Revised UCLA Loneliness Scale^¶^ [73]		X		X	X
Perceived Stress Scale^¶^ [74]		X		X	X
Patient Health Questionnaire – 8^¶^ [75]		X		X	X
Generalized Anxiety Disorder – 7^¶^ [76]		X		X	X
Veterans RAND 12 Item Health Survey^¶^ [77]		X		X	X
Patient-Reported Outcomes Measurement Information System (PROMIS) Sleep Disturbance^¶^ [78]		X		X	X
AIDS Clinical Trials Group Falls Questionnaire^¶^ [79]		X		X	X
Goal Systems Assessment Battery – Alcohol^†,††^ [41, 42]		X		X	X
Readiness to Change Questionnaire – Alcohol^††^ [40]		X		X	X
Phosphatidylethanol (PEth) [56]					X
Ecological Momentary Assessment (15 days)		X		X	
Treatment Satisfaction [80]				X	
Treatment Utilization					X

^†^, alcohol and other substance use outcomes

^§^, pain outcomes

^¶^, physical and mental health

^††^, potential mediators and moderators of behavior change for pain and alcohol use

### Study conditions

#### Intervention

##### Rationale for integrated videoconferencing intervention

There would be considerable benefit to have an integrated approach to treat unhealthy alcohol use and pain for utilization across a variety of health care and community settings. Self-management interventions grounded in CBT have proven efficacious in reducing alcohol use and chronic pain across a wide range of populations [81, 82]. In the context of chronic medical conditions, these approaches emphasize strategies for addressing motivation to change and developing cognitive-behavioral skills for initiating and maintaining change [83]. Integrated interventions provide the opportunity to condense the number of sessions through common underlying treatment targets (e.g., stress coping) as well as helping patients understand the associations between pain and alcohol use in their own lives.

Telehealth through a videoconferencing platform represents an increasingly available method of intervention delivery that can enhance treatment access and adherence [84]. Telehealth has proven particularly advantageous among populations facing significant barriers to treatment, such as non-urban and low-income individuals, and has gained similar levels of treatment satisfaction compared to in-person treatment [85–89]. Second, the web-based formatting facilitates patient connection to supplemental materials (e.g., web-based assessments, EMA, video-skills training) that may extend interventions [84, 85]. Overall, this approach provides greater flexibility in terms of how and when interventions may be delivered.

##### INTV structure and content

The INTV utilizes a self-regulation framework [90] to integrate evidence-based approaches for chronic pain and unhealthy drinking. Specifically, INTV integrates Motivational Interviewing (MI; [91]) and cognitive-behavioral skill training [92, 93] for unhealthy drinking with cognitive-behavioral and self-management approaches for chronic pain [82, 94]. These approaches have proven efficacious for reducing unhealthy drinking and chronic pain as individual treatment targets among PLWH [95, 96]. The central goals of INTV are to increase motivation and self-efficacy to change and provide cognitive and behavioral skills to manage pain and reduce alcohol use. The intervention will be delivered through internet-based videoconferencing and supplemented with web-based content. The initial treatment session provides a rationale for addressing alcohol and pain together in the context of HIV management, and initiates MI related to alcohol use. Over the subsequent weeks, participants receive 6 additional treatment sessions, each lasting 45–60 min, for a total of 7 sessions (see Table 2). The intervention content was adapted for PLWH through a mixed methods approach and underwent preliminary testing to assess feasibility and acceptability [95, 96].

**Table 2:** Overview of intervention content

Session #	Brief Content Description
Session 1	Introduction to Intervention: Provide psychoeducation about alcohol and pain, deliver brief motivational intervention for alcohol, explain rationale for intervention approach, set initial goals, and review technology requirements.
Session 2	Behavioral Activation: increasing pleasant events, considering personal life goals and the role of alcohol.
Session 3	Identifying triggers of pain and factors that reduce pain; review diaphragmatic breathing, progressive muscle relaxation, imagery, and self-monitoring.
Session 4	Identifying unhelpful (automatic) thoughts and beliefs; review cognitive restructuring, managing negative thoughts related to stress, and the relationship between negative affect and pain
Session 5	Review planning, time-based pacing, and alcohol-related coping skills, with emphasis on harm reduction skills
Session 6	Discuss sleep hygiene and the influence of lifestyle factors on pain and stress
Session 7	Review of skills; discuss continuing self-management beyond treatment and successes, challenges, and barriers.

*Note*: https://sites.bu.edu/mhealth/ contains supplementary material accompanying each session

#### Control

Participants randomized to CTL will meet once with an interventionist (“health counselor”) via videoconferencing for 20–30 min and receive psychoeducation about the effects of alcohol and pain on HIV symptoms, recommendations for alcohol use reduction, and a list of treatment resources for alcohol and chronic pain. CTL participants complete the same research assessments as those in INTV.

##### Rationale for control condition

The choice of this control condition was based on our goal to obtain an estimate for the impact of INTV compared to the current standard of care. We believe that psychoeducation and brief advice serves as an optimal control condition at this stage of intervention efficacy testing because it provides a test of whether the proposed intervention provides benefit compared to the type of intervention that may be commonly provided as part of an HIV-care visit (treatment-as-usual) or community setting. We recognize that different control conditions offer different types of information relevant to the efficacy and effectiveness of an intervention and that alternative control conditions (e.g., weekly contact) may be utilized in the next stages of research should this approach prove to demonstrate evidence of efficacy.

### Interventionist training, Supervision, and Fidelity Assessment

Interventionists will be clinical psychologists and advanced level PhD clinical psychology students trained in both INTV and CTL condition content. Training will include MI in medical settings, [91] CBT for pain, [94] and alcohol self-management [92, 93]. Interventionists will participate in trainings on the INTV manual, supplemented by video recordings, role plays, and structured training cases. Supervision will occur on a biweekly basis, during which individual cases will be discussed. Interventionists also will conduct post-intervention fidelity assessments. Sessions will be digitally recorded with participant consent and select cases may be reviewed by interventionists and supervisors as needed.

### Statistical design and analysis

#### Sample size determination

A sample size of 250 (*n* = 125 to each condition) will be randomized. With a conservative estimate of a 20% attrition rate by 6-month follow-up, this sample size gives power (80%) to detect medium effects (Cohen’s d = 0.40) at the 2-tailed significance level (0.05) for INTV versus CTL at 6-month follow-up on both pain and alcohol outcomes. The anticipated effect size is based on the following: (1) meta-analyses of CBT interventions compared to assessment and minimal interventions suggesting medium post-intervention effects on alcohol and pain; [98–100] and (2) results of our pilot study which showed medium-to-large pre-to-post changes (Cohen’s d from 0.79 to 1.0) within participants receiving INTV [95, 96]. Moreover, the availability of repeated measurements (to include a baseline covariate) increases statistical power to detect intervention effects for the analysis.

#### Primary and secondary analyses

Descriptive statistics will be provided for the baseline characteristics of the full sample and by condition (i.e., INTV vs. CTL). Multiple linear regression analyses will be used to test whether those in the INTV group consume fewer drinks per week, engage in fewer episodes of heavy episodic drinking, and report lower PEG ratings than those in the CTL group at 6-month outcome. Covariates will include age, sex at birth, and corresponding baseline measures of alcohol use and pain. Secondary analyses will examine related outcomes (i.e. alcohol related consequences, pain intensity, pain severity) using a parallel approach. Exploratory analyses will examine INTV effects on outcomes including substance use frequency, depressive symptoms, anxiety symptoms, HIV symptom index scores, HIV risk-taking behaviors, mental functioning, and physical functioning at 6 months using multivariate regression models. For PEth analyses, we will use the lower limit of quantification as a cut-off of > 50 as a measure of unhealthy alcohol use and a cutoff of PEth > 8 ng/ml) as a marker of any alcohol use. Logistic multiple regression with the age and race/ethnicity will be used to examine intervention effects on 6-month PEth outcomes [101]. Secondary aims will be addressed, in part, by using baseline interview measures of readiness-to-change (for alcohol) and baseline GSAB questionnaire ratings of self-efficacy and planning (for both alcohol and pain outcomes). Planned covariates include those described under primary aims and will include baseline values of the questionnaire measure being evaluated. The Condition x Moderator term will be added to the full models to examine interaction effects, which will be followed with simple slopes analyses to determine the direction of effects.

#### EMA data analysis

Consistent with previous work using a highly individualized approach [102] to treatment and assessment, self-regulatory responses from EMA periods will also be used to conduct moderation and mediation analyses. Moderation analyses will test the extent to which daily ratings of readiness-to-change, alcohol coping self-efficacy, and alcohol-related planning, moderate the effect of the INTV intervention on alcohol use. Mediational analyses will test the extent to which the intervention reduces both alcohol use and chronic pain through changes in self-regulatory processes when exposed to relevant contexts. Analyses will use aggregated scores from events in which the participant indicates that they were exposed to relevant contexts, including moderate or greater pain, or the opportunity to use alcohol [102]. As with the questionnaire data, we will use structural equation modeling (SEM) to test the hypothesis that increases in context-specific self-efficacy and planning will mediate the relationship between condition (INTV vs. CTL) and 6-month outcomes. Bias-corrected bootstrap confidence interval estimates will be used to test the mediation hypotheses [103]. As described, power of > 80% is estimated when the standardized effect of path a and path b represents a small-to-medium effect. Covariates in the EMA analyses will include the number of event monitoring events completed, as well as the number of relevant pain or drinking opportunity events identified.

EMA data will also contribute to several exploratory analyses which address event level questions, including how the intervention influences associations between cues (e.g., mood) and pain and alcohol use outcomes. It is hypothesized that, when day is included as a covariate to control for potential systematic changes over time, higher negative mood earlier in the day will be associated with higher chronic pain or unhealthy drinking later in the day. Within-subject associations between mood and chronic pain or unhealthy drinking may vary by treatment group, and it is hypothesized that those randomized to INTV will demonstrate attenuated associations relative to the CTL group.

## Discussion

Unhealthy drinking and chronic pain are common comorbidities among PLWH that have reciprocal influences on one another and negatively impact HIV outcomes. The development of an efficacious, integrated, telehealth approach is an important step toward addressing treatment uptake challenges common in these comorbid conditions. This protocol describes an RCT for PLWH with co-occurring unhealthy alcohol use and chronic pain. The INTV telehealth intervention integrates MI and cognitive-behavioral skill training to target unhealthy drinking and cognitive-behavioral self-management approaches for chronic pain management. EMA before and after intervention delivery allows for identification of moderators and mediators of the intervention effect.

Although this protocol makes an important contribution to the literature, it has limitations. The intervention aims to improve access and utilization of treatments for pain and unhealthy drinking, however, the recruitment and enrollment procedures for this study occur online and therefore require internet access. Moreover, inclusion criteria also require that participants own a smartphone or other device (e.g., tablet, computer) with internet access to allow for videoconferencing with the health counselor and completion EMA surveys. Individuals who do not have internet access are therefore unable to engage with the study advertisements or enroll to participate. Similarly, those who are unable to provide a contact may not participate. These criteria may reduce the generalizability of findings. Finally, blood collection for PEth analysis is both self-administered and requires mailing the sample to the laboratory. Although this procedure is designed to increase the capacity to assess PEth with a national sample, its feasibility among this population is largely unknown.

Notwithstanding these limitations, the INTV trial will test the efficacy of a novel, integrated telehealth intervention for chronic pain and unhealthy drinking. This integrated video telehealth program has the potential to address structural barriers to the reduction of chronic pain and unhealthy drinking among PLWH.

## Data Availability

Data collected for this study will be submitted to the NIAAA Data Archive (NIAAA DA) repository. Data will be de-identified, individual-level data. To facilitate data sharing, we plan to use data collection instruments/data dictionaries that have already been defined rather than create new versions of those data dictionaries, whenever possible. We will also provide the required supporting documentation when sharing our data, including assessment clinical trial protocol, schedule of visits, manual of operations, case report forms/assessment instruments, and analytic/statistical algorithms used to derive variables in publications. We will adhere to all required data collection instruments for data harmonization purposes, and all subjects in the dataset will have a global unique identifier (GUIDs). We will link any relevant publications to our study in the Data Archive.
